# Supplementary material for: Mycotoxin DON Accumulation in Wheat Grains Caused by Fusarium Head Blight Are Significantly Subjected to Inoculation Methods
Source: Toxins (Basel). 2022 Jun 15;14(6):409. doi: 10.3390/toxins14060409 (PMC9229350; doi:10.3390/toxins14060409)
Supplement: Supplementary file 1 [file toxins-14-00409-s001.zip › toxins-1759806-supplementary.pdf]

# Supplementary Materials: Mycotoxin DON Accumulation in Wheat Grains Caused by Fusarium Head Blight Are Significantly Subjected to Inoculation Methods

**Limei Xian, Yuhui Zhang, Yi Hu, Suqin Zhu, Zhuo Wen, Chen Hua, Lei Li, Zhengxi Sun and Tao Li**

**Table S1.** Variation of PSS, DON content in grains and PPSD in the *Fhb1* near-isogenic lines.

| Inoculation method | Year | PSS | | | | DON content in grains (μg·kg$^{-1}$) | | | | PPSD | | | |
|---|---|---|---|---|---|---|---|---|---|---|---|---|---|
| | | Max | Min | Mean | Error | Max | Min | Mean | Error | Max | Min | Mean | Error |
| UBFI | 2020 | 0.90 | 0.18 | 0.35 | 0.17 | 4224.38 | 103.43 | 1221.76 | 1345.20 | | | | |
| | 2021 | 0.92 | 0.38 | 0.67 | 0.18 | 2676.14 | 528.49 | 1527.91 | 654.44 | — | | | |
| | BLUP | 0.76 | 0.36 | 0.51 | 0.10 | 2651.56 | 795.80 | 1448.56 | 557.26 | | | | |
| BBFI | 2020 | 1.00 | 0.27 | 0.85 | 0.25 | 681.53 | 42.85 | 175.90 | 118.41 | 1.00 | 0.15 | 0.84 | 0.25 |
| | 2021 | 1.00 | 0.16 | 0.74 | 0.26 | 1262.59 | 541.85 | 798.51 | 186.88 | 1.00 | 0.00 | 0.62 | 0.34 |
| | BLUP | 0.98 | 0.26 | 0.78 | 0.23 | 490.71 | 490.71 | 490.71 | 0.00 | 0.98 | 0.12 | 0.71 | 0.27 |
| BRII | 2020 | 0.60 | 0.01 | 0.22 | 0.20 | 857.47 | 37.81 | 173.60 | 181.49 | 0.61 | 0.00 | 0.13 | 0.19 |
| | 2021 | 0.46 | 0.00 | 0.12 | 0.12 | 663.41 | 187.37 | 334.80 | 130.96 | 0.17 | 0.00 | 0.02 | 0.05 |
| | BLUP | 0.47 | 0.04 | 0.19 | 0.13 | 283.11 | 256.62 | 265.12 | 7.61 | 0.15 | 0.06 | 0.08 | 0.03 |

PSS—proportion of symptomatic spikelets; DON—deoxynivalenol; PPSD—proportion of premature spike death; UBFI—upper bilateral floret injection; BBFI—basal bilateral floret injection; BRII—basal rachis internode injection; BLUP— best linear unbiased prediction.

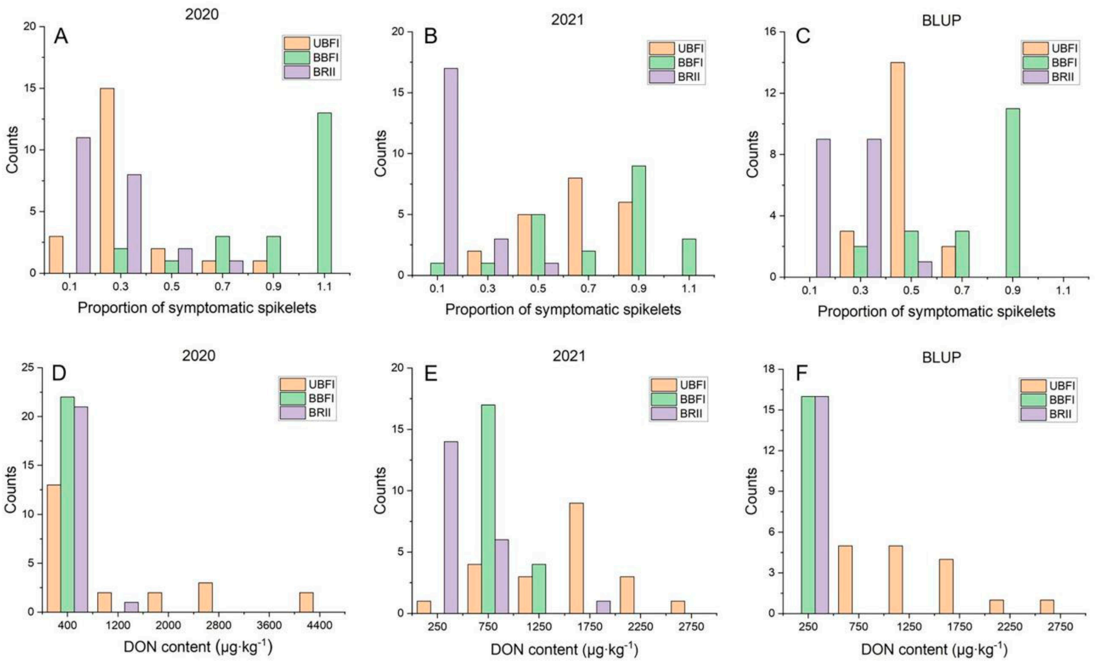


**Figure S1.** Distribution of proportion of symptomatic spikelets (PSS) and deoxynivalenol (DON) content in the *Fhb1* near-isogenic lines under the three inoculation methods. (A, D) Year 2020. (B, E) Year 2021. (C, F) Predicted PSS and DON content by best linear unbiased prediction (BLUP).

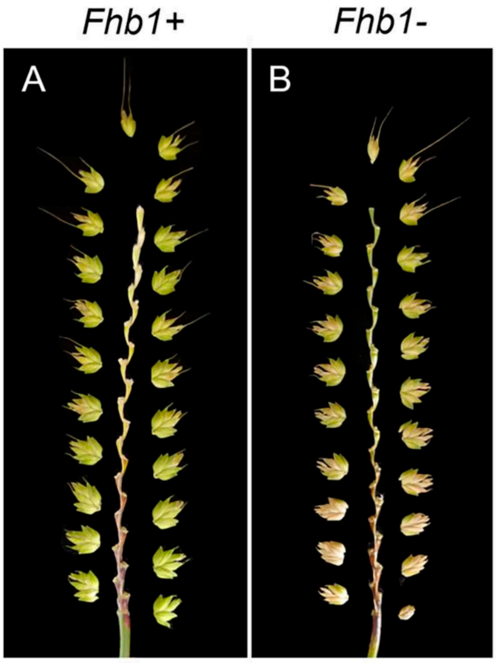


**Figure S2.** The rachis and spikelets of the wheat spikes inoculated by basal rachis internode injection (BRII). (A) The rachis of a wheat spike carrying *Fhb1* showed obvious bleaching, but no symptomatic spikelets were observed. (B) The rachis of a wheat spike without *Fhb1* showed obvious bleaching, and the symptomatic spikelets were observed.

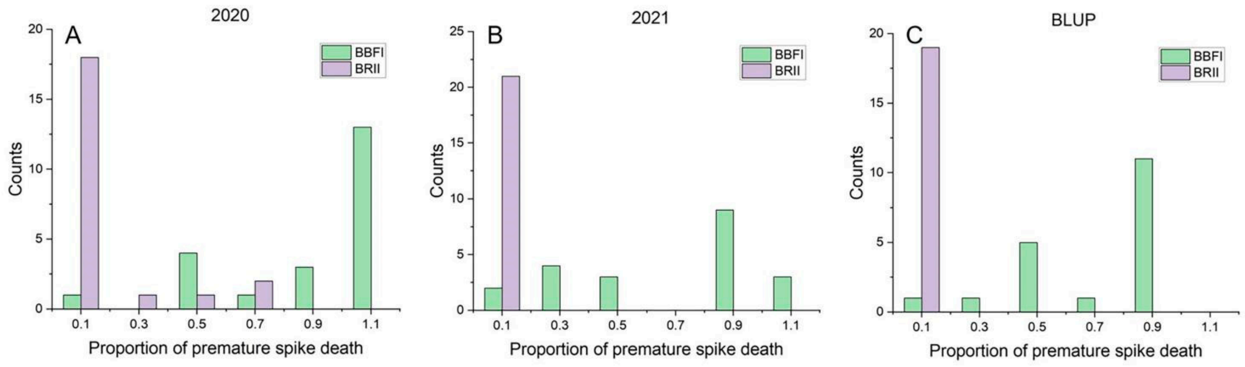


**Figure S3.** Distribution of proportion of premature spike death (PPSD) in the *Fhb1* near-isogenic lines under the methods of basal bilateral floret injection (BBFI) and basal rachis internode injection (BRII). (A) Year 2020. (B) Year 2021. (C) Predicted PPSD by best linear unbiased prediction (BLUP).
